# The American Academy of Dental Science

**Published:** 1868-01

**Authors:** 


### EDITORIAL DEPARTMENT.

---

*The American Academy of Dental Science.*—A number of prominent dental practitioners in Boston have established an institution, to which they have given the above title.

Their object is set forth in the following preamble of the Constitution and By-Laws, a copy of which we have received from the Corresponding Secretary.

"In view of the great importance of the art of Dentistry to the human race, and for the purpose of promoting the theoretical and practical knowledge of the same, as well as for elevating the standard of the science, and the requirements of practitioners of the profession, we, the undersigned, do hereby agree to establish an institution for cultivating and advancing a knowledge of Dentistry among its members." The officers for the coming year are; President, E. T. Wilson, M.D.; Vice President, D. M. Parker, M.D.; Recording and Corresponding Secretary, E. N. Harris, D.D.S.; Treasurer, J. L. Williams, M.D; Librarian, John Clough, M.D.; Board of Censors, E. G. Tucker, M.D., D. M. Parker, M.D., J. L. Williams, M.D. From the high professional character of the officers of this new institution, we are confident that it will exert great influence in accomplishing of the object for which it has been instituted.

