# Typographical Error

**Published:** 1868-02

**Authors:** 


*Typographical Error.*—The word "of" in second line from end of Editorial on *American Academy of Dental Science* in January No. of Journal is superfluous.

